# Detection of Unfocused EEG Epochs by the Application of Machine Learning Algorithm

**DOI:** 10.3390/s24154829

**Published:** 2024-07-25

**Authors:** Rafia Akhter, Fred R. Beyette

**Affiliations:** Department of ECE, College of Engineering, University of Georgia, Athens, GA 30602, USA; rafia.akhter@uga.edu

**Keywords:** electroencephalography, distraction, artifacts, machine learning algorithm, oddball paradigm, human visual inspection, EEGLab

## Abstract

Electroencephalography (EEG) is a non-invasive method used to track human brain activity over time. The time-locked EEG to an external event is known as event-related potential (ERP). ERP can be a biomarker of human perception and other cognitive processes. The success of ERP research depends on the laboratory conditions and attentiveness of the test subjects. Specifically, the inability to control experimental variables has reduced ERP research in the real world. This study collected EEG data under various experimental circumstances within an auditory oddball paradigm experiment to enable the use of ERP as an active biomarker in normal laboratory conditions. Then, ERP epochs were analyzed to identify unfocused epochs, affected by typical artifacts and external distortion. For the initial comparison, the ability of four unsupervised machine learning algorithms (MLAs) was evaluated to identify unfocused epochs. Then, their accuracy was compared with the human inspection and a current EEG analysis tool (EEGLab). All four MLAs were typically 95–100% accurate. In summary, our analysis finds that humans might miss subtle differences in the regular ERP patterns, but MLAs could efficiently identify those. Thus, our analysis suggests that unsupervised MLAs perform better for detecting unfocused ERP epochs compared with the other two standard methods.

## 1. Introduction

Electroencephalography (EEG) is a non-invasive and continuous method that is used to monitor and analyze human brain activity. The synchronized capture of EEG in response to external stimuli is termed event-related potential (ERP). ERPs provide a detailed view of brain responses to specific sensory stimuli, offering high temporal resolution [1] and potential applications as biomarkers for perception, behaviors, and cognitive processes [2,3]. While ERPs have been effective in controlled laboratory environments, their success relies on strict control over experimental conditions, including minimizing external electrical noise and distractions and ensuring sustained attention [4]. Challenges arise when experimental conditions cannot be tightly regulated, limiting the application of ERP research in less controlled settings [5].

The EEG signal comprises a combination of various activities originating from both neural and non-neural sources. These non-neural signals are also known as artifacts. These artifacts can distort the EEG, reducing the signal-to-noise ratio (SNR) in ERP experiments and potentially leading to inaccurate interpretations of experimental effects [6]. Therefore, it is crucial to eliminate artifacts from recorded EEG data before proceeding to the final analysis of ERP data. In general, the ideal ERP experiments are conducted in a laboratory environment that eliminates electrical noise sources and includes careful task instructions that are designed to maximize test subject attentiveness while simultaneously minimizing test subject actions that produce classical artifacts. The failure to adhere to experimental practices that approach the ideal ERP experiment results in a reduced SNR, which reduces the confidence levels in the repeatability of experimental results (i.e., *p* values) and ultimately can lead to the erroneous interpretation of experimental outcomes. As a result, the standard data processing approach in ERP experiments is to identify and remove epochs that deviate from the normal ERP response to an experimental procedure. As such, it is only necessary to identify abnormal epochs (i.e., epochs that have been corrupted by artifacts). It is not necessary to differentiate different root causes that produce abnormal epochs. This is true because regardless of what causes an abnormal epoch, the response to identifying an epoch as abnormal is to remove the abnormal epoch from subsequent data analysis methods. Thus, if there is a method that could differentiate abnormal epochs from normal epochs, then it could improve the reliability of the ERP result. Further, if the abnormal/normal differentiation process worked on data that were collected outside of a carefully controlled lab setting, then the use of ERP signals could be enabled as a biomarker that can be quantified while the test subject is functioning in the real world (i.e., outside of the carefully controlled laboratory environment).

This process has been labor-intensive and manual, making it impractical for real-time ERP signal capture. This project aims to address this challenge by introducing machine learning models capable of automatically screening ERP data and identifying and rejecting data epochs affected by poor signal quality, measurement artifacts, or subject distraction or inattention. In this research, we developed an experimental framework for screening and recording EEG data within an uncontrolled laboratory environment, where no deliberate efforts were made to minimize electronic noise or mitigate factors contributing to EEG/ERP data collection artifacts. Through this setup, we observed that unsupervised machine learning algorithms (MLAs) offer highly effective means of identifying artifacts, particularly those stemming from eye blinks and body movements. Furthermore, we discovered that MLAs can also detect unlabeled artifacts caused by both auditory and visual distractions. Notably, the DBScan (density-based spatial clustering of applications) algorithm emerged as the most efficient method for detecting unlabeled artifacts. Moreover, we observed that ERP datasets can exhibit improved cleanliness under uncontrolled laboratory conditions, simplifying data processing and thereby enhancing the quality of EEG investigations.

## 2. Background and Literature Review

In 1949, Shannon initially presented the theory for removing artifacts from recorded EEG signals [7]. Since then, EEG-based research has seen considerable progress, with artifact removal remaining a crucial step in data processing. Various hardware and software methods have been developed for this purpose to address specific types of artifacts. For instance, in detecting eye blinks or movements, electrooculography (EOG) electrodes are commonly utilized [8]. These electrodes are placed near the eyes to capture signals associated with these movements [9]. However, integrating EOG channels alongside multiple EEG channels can result in substantial increases in data volume, posing challenges for data management. Moreover, certain signal processing techniques for artifact recognition may necessitate scalp maps and independent component analysis. These methods often demand significant computing resources and may require extended processing times. Additionally, to ensure accurate artifact removal, experiments are typically conducted in laboratory environments, which are designed to minimize external sources of electronic noise.

When employing software-based artifact identification and removal, lengthy preprocessing steps are often needed, complicating the processing of ERP data in real time, especially because human involvement is frequently essential for labeling the artifacts. Yet, human involvement in this process can introduce errors due to the tedious and time-consuming nature of visually inspecting EEG/ERP data [10]. Even with variations in artifacts depending on factors like participants, tasks, or experimental protocols, relying on human intervention for artifact identification/rejection can yield questionable results. As a result, in recent years, considerable efforts have been focused on integrating artificial intelligence approaches into EEG/ERP data analysis.

In 2017, Raduntz and colleagues [11] introduced a technique for automatically removing artifacts, using a machine learning technique, trained on structures derived from independently analyzed components. They evaluated the classifier performance based on visual experiments. The topographies employed encompassed filtered topographic plots, power spectra, and an artificial neural network. In the meantime, in 2019, Agarwal et al. anticipated an unsupervised method for detecting artifacts (ex., eye blink) and found an accuracy of 98.15% [12]. They utilized the EEG capture system, OpenBCI, and EEGLab for data analysis, with participants performing reading and video-watching tasks while EEG data were recorded. Besides EEGLAB, they compared their eye blink detection techniques with SVM (Support Vector Machine) [13] and k-NN (k-nearest neighbor) learning-based strategies [14], yielding a relatively lower accuracy of 46.49% and 67.82%, respectively.

To improve classifier efficacy, in 2020, authors Lee et al. developed a deep learning approach, joining attention modules with the Bayesian approach [15]. They adapted the method of ASR (artifact subspace reconstruction) to eliminate line noise post-filtering and address artifacts related to eye blinks and lateral eye movements, which are extensively distributed across the human scalp, showing substantial variability [16]. Despite achieving high classification accuracy, this method is not easy to use. Because it requires human involvement, experimental methods related to the task and a module for measuring attention are required.

In 2021, authors Sadiya et al. described a technique for reducing the noise of EEG data. Their method for detecting artifacts was a task-dependent experiment and the correction procedure was learning-based. Also, they extracted a lot of features (58) from the signals based on predefined objectives [17].

## 3. ERP Technique and Artifact

### 3.1. ERP Technique

In EEG studies, a widely used ERP technique is the oddball paradigm. In this paradigm, participants are exposed to a repetitive sequence of one stimulus and occasionally interrupted by a second, less frequent stimulus. The response to this unexpected stimulus is recorded and identified as an “oddball”. In 1975, Squires et al. conducted the initial application of this experimental approach in EEG research [18], focusing on an auditory version of this classic ERP experiment, in contrast to the visual stimulus variant.

**Figure 1:**
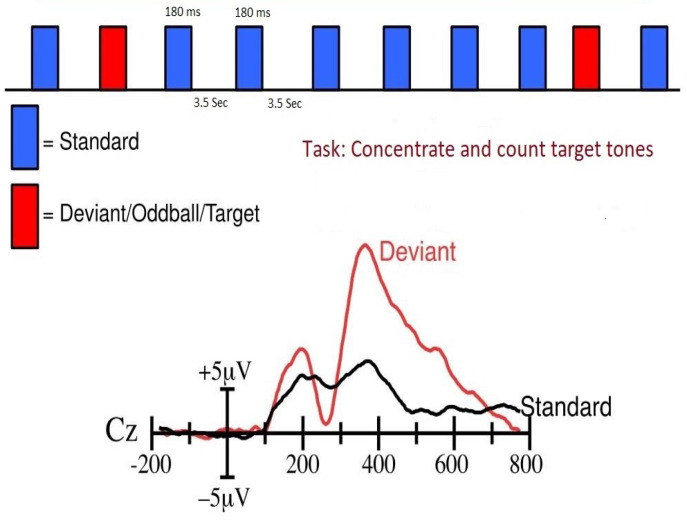
Event-related potential. The upper picture shows the common (standard) events in blue and the odd (deviant) events in red. It also shows for odd events that the ERP peak is higher [22].

Figure 1 shows the common and uncommon stimuli sequence (top) and ERP for a visual experiment (down). The ratio of common and uncommon stimuli is 80:20. The stimuli, which are blue-colored, are common stimuli and red stimuli are uncommon/odd stimuli. In any oddball paradigm experiment, the test subjects are told to concentrate on uncommon stimuli and try to ignore common stimuli. Then, averages for both stimuli are generated, which are known as the ERP. From Figure 1, it is shown that both the ERP peaks around 300 ms and the ERP peak for uncommon stimuli (red) are higher compared to the common ERP (black) [19,20]. The components that peak around 300 ms are known as P300 or P3 components. ERP has various components that are used in EEG research, but the P300 component is one of the most common components. The most important reason is that it is easily identifiable due to its high peak compared to others [21].

### 3.2. Artifacts

Human brain signals are the blend of both neural and non-neural sources of action. The activities that are non-neural are unwanted in the EEG experiments and are classified as anomalies or EEG artifacts. Their reason might be a loss of attention, body movements, eye movements, other electrical signals, etc. Table 1 shows a list of sources of anomalies or artifacts during EEG/ERP signal recording [23].

## 4. Artifact Detection by Machine Learning Application

In EEG data, artifacts are those points that are different from other regular points. These unusual points are similar to an “outlier” in a dataset. An outlier is defined as any data point or object that significantly diverges from the rest of the dataset [25,26]. Outliers are often disregarded in data mining as anomalies or mere noise. Unlike the conventional approach of classification, where a trained classifier is used for the assessment of other test data, anomaly detection encompasses various setups. The setup for anomaly data selection depends on the performance of the EEG indicators in the dataset. There are three types of detection techniques:

i. Supervised methods;
ii. Semi-Supervised methods;
iii. Un-Supervised methods.

### 4.1. Supervised Method

**Figure 2:**
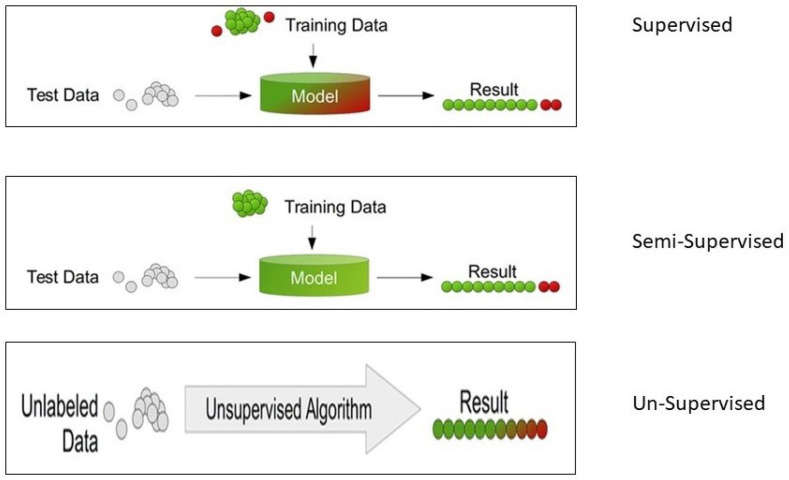
Machine learning (ML) techniques for detecting unusual data points [30]. In the figure, green balls are regular data, red balls are unusual data and grey balls are test data.

Supervised methods require two distinct datasets: training and test datasets. The training datasets are normally labeled as required for the learning process. For example, in the artifact detection method, they will be labeled as “regular” and “unusual” datasets. The model first trains with a training dataset and is tested with other test datasets. As the method already learned by the training dataset, the model can distinguish the data points from the unseen datasets and return the data points as regular or unusual data points [27,28,29]. Figure 2 (top) shows the process in brief.

### 4.2. Semi-Supervised Method

This method requires both test and training datasets, and the training dataset must not contain any unusual datasets. All the data must be neural data, so that while testing, any data that are not matched with training data characteristics will be classified as an unusual or artifact-mixed dataset [31,32,33]. Figure 2 (middle) shows the process in brief.

### 4.3. Un-Supervised Method

The unsupervised method for the detection of artifacts is highly adjustable because it does not necessitate manual labeling of the training data and does not differentiate between training and test datasets. This approach relies solely on assessing the data based on its intrinsic characteristics. To distinguish between normal and outlier data, methods such as distances or densities are often utilized for estimation. Figure 2 (bottom) shows the process in brief. This article exclusively focuses on the method of the unsupervised technique for the detection of these non-neural activities or artifacts in the EEG recording. The assumption is that the majority of the recorded EEG data are neural, with a lesser amount of the signal being unusual. These unusual signals are considered unfocused and artifact-mixed [34,35].

## 5. Experimental Procedure and Data Preprocessing

### 5.1. Dataset

**Figure 3:**
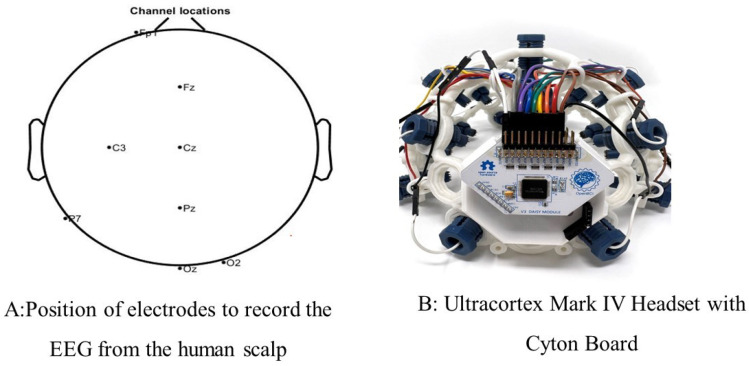
The electrode positions on the human scalp for the EEG recording is shown in subfigure (**A**). The positions are based on the International 10/20 system. (**B**) shows the Ultracortex Mark IV headset with a cyton board installed. This was used for recording EEG [36].

In this study, EEG data were recorded from 25 test subjects (male/female), and all the subjects were adults, with a minimum age of 18 (no more than 40). Before recording the EEG data, they consented that they were physically and mentally stable with no hearing problems. The Institutional Review Board (IRB) of the University of Georgia approved this study before the data were recorded. All the data were recorded in the lab at the UGA. While recording the EEG data, the test subjects wore a Mark IV Ultracortex headset where 8 electrodes were positioned for the recording [36,37,38]. The location of the electrodes was designed based on the international 10–20 system. This system uses dry electrodes for recording EEG from the scalp of humans. The position of the 8th electrode is located on the “Frontal, central, parietal, and occipital” area of the human scalp. The electrode positions (Fp1, Fz, C3, Cz, P7, Pz, O2, and Oz) are shown in Figure 3A, and the Ultracortex headset is shown in Figure 3B.

This EEG experiment comprised four setups, and for every test subject, there were four datasets. Every setup contained 50 auditory stimuli. So, in total, there were 25 × 4 × 50 = 5000 auditory stimuli for the analysis. For the EEG recording, an Open-BCI cyton board was also used. It had 5 input–output (IO) pins, which are capable of dealing with both analog and digital signals. In this experiment, 2 digital pins were used, which converted an auditory tone to a digital sequence to identify events. In Figure 3B, it is shown that the cyton board is attached to the Ultracortex headset. The auditory stimuli were generated by the use of Micromedia and the PIC24EP board, which are available from MikroElectronika Inc. (Belgrade, Serbia) [39].

### 5.2. Experimental Procedure

**Figure 4:**
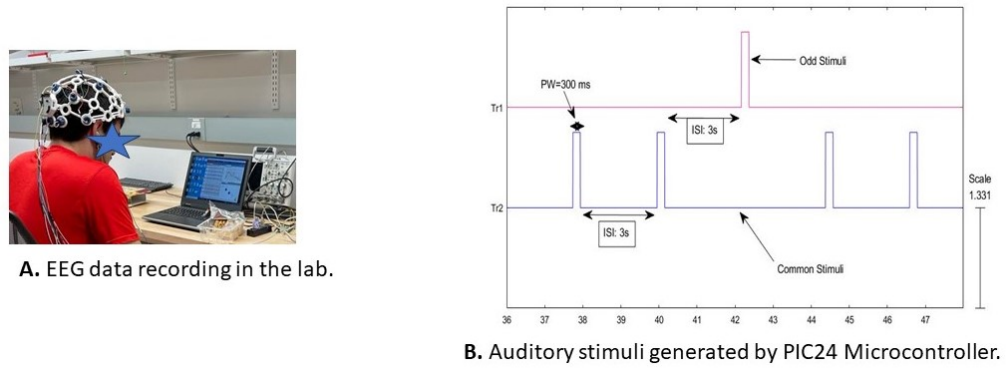
The recording of EEG data from a Test Subject in the lab is shown in (**A**). The auditory stimuli generated by the PIC24 microcontroller is shown in (**B**). T1 shows odd and T2 shows common stimuli. The tone duration is shown as PW = 300 ms and the ISI (interstimulus interval) is shown as 3 s. For every setup, there were 50 auditory stimuli and the test subject heard 10 odd stimuli and 40 common stimuli.

While recording the EEG data, the test subjects were sitting in a comfortable chair, wearing a Mark IV Ultracortex headset. There was a laptop in front of the test subject, and at the same time, they were hearing auditory stimuli. Figure 4A shows the setup for the experiment while the data were recorded from a test subject in the lab. There were 4 experimental setups, and in every setup, the test subject heard 50 auditory stimuli, 40 common ones (1000 Hz), and 10 odd stimuli (2000 Hz). The auditory stimuli, generated by the PIC24 microcontroller, are shown in Figure 4B. The audio volume was set around 70 dB, which is slightly louder than normal conversation. Each auditory stimulus duration was 300 ms and the gap between two stimuli (ISI: interstimulus interval) was 3 s.

In the first step, the test subject was not instructed to do anything except to hear the stimuli. This condition is termed “mind wandering” and the dataset is named as the “mind-wandering dataset”. In the 2nd step of the experiment, the subject was instructed to count the odd stimuli, and at the same time, the test subject was watching a video without any sound. From this step, the effect of visual distraction on the EEG data would be analyzed. This is one of the test datasets. This step is known as “visual”. In the 3rd step in Table 2, the same sequence was repeated, but this time, the video was shown with sound. This step is known as “audio–visual” distraction and this dataset is another test dataset. Finally, the test subjects were instructed to concentrate and count the odd stimuli. They were also instructed to give their maximum effort by not moving their body or head, even trying not to blink their eyes, so that there should be no artifacts or data with the minimum artifacts possible could be recorded. This condition is known as the “ideal or gold standard” and the dataset recorded from these steps is known as the “ideal” dataset. Table 2 shows the full experimental setup for the EEG recording. Every experimental setup took 3–4 min and there was a 4–5 min gap between all the experimental steps. Overall, it took around 35 min to 45 min for a full experiment with all 4 steps with every test subject.

### 5.3. Data Preprocessing

In cognitive experiments, the use of the filter is very important to reduce noise. Specially, for line noise, a 60 Hz filter is used (in the USA). In general, low-pass filters are commonly employed to diminish noise from electrical lines and EMG, whereas the use of high-pass filters eradicates steady changes in voltage attributable to skin potentials and other slow variations in the voltage offset, thereby enhancing the arithmetical power of the outcomes. Most of the relevant portion of the ERP waveform in a typical cognitive neuroscience experiment consists of frequencies of 0.1Hz and 30 Hz [40]. In EEG signal processing, high-pass filters are often applied to remove the baseline drift caused by electrode impedance while preserving neural oscillations and event-related potentials (ERPs) in the higher-frequency bands. It is also recommended in various research to use 0.1–1 Hz of an HPF. However, it is found that filtering with a high cut-off frequency (around 0.5 or more) may decrease the statistical power of the ERP waveform. In our experiment, we found strong signals with less distortion by 0.3 Hz. Finally, we applied a 0.3 Hz HPF and a 60 Hz stop-band filter for the processing of the EEG data. In addition, a 7-point moving average filter (MAF) was used to remove unwanted artifact-related noise after obtaining the ERP for both the common and odd stimuli. The MAF is very commonly used in ERP studies to remove unwanted peaks for noise.

## 6. Practical Implementation

Table 3 shows the different methods for analyzing the recorded EEG data, which were collected from the four types of experimental methods, as described in Table 2. For the identification of unfocused or artifact-corrupted ERP epochs, we applied three methods.

They are as follows:

i. Human visual inspection (HVI);
ii. The application of the EEGLab Toolkit;
iii. The application of machine learning algorithms.

Dataset G was collected (Table 3, column 2) in data collection step 4, which is termed an ideal or gold-standard condition. In this step, the test subject made their maximum effort to concentrate on the experiment. This G dataset was inspected by humans who marked the ERP epochs as either regular or unfocused and/or artifact-corrupted. Dataset T1, collected from step 1 (column 3), is a mind-wandering dataset where test subjects did not make any effort to reduce artifacts. Dataset T2 (column 4), collected from step 2, is known as an unfocused test dataset as we intentionally introduced visual distraction, and dataset T3 (column 5) was collected from step 3. This dataset is also an unfocused test dataset as here both audio and visual distraction were introduced. In Test Method 1, the identification of unfocused ERP epochs was performed by HVI, and after identification, datasets T1, T2, and T3 were termed as T1H, T2H, and T3H. In Test Method 2, the EEGLab Toolkit was used for the identification of unfocused ERP epochs from the same T1, T2, and T3 datasets, and after being identified, they were termed as T1E, T2E, and T3E. Finally, in Test Method 3, four types of unsupervised MLAs were applied for the identification of unfocused ERP epochs from the same T1, T2, and T3 datasets. After identification, the datasets were termed T1M, T2M, and T3M.

### 6.1. Human Visual Inspection (HVI)

**Figure 5:**
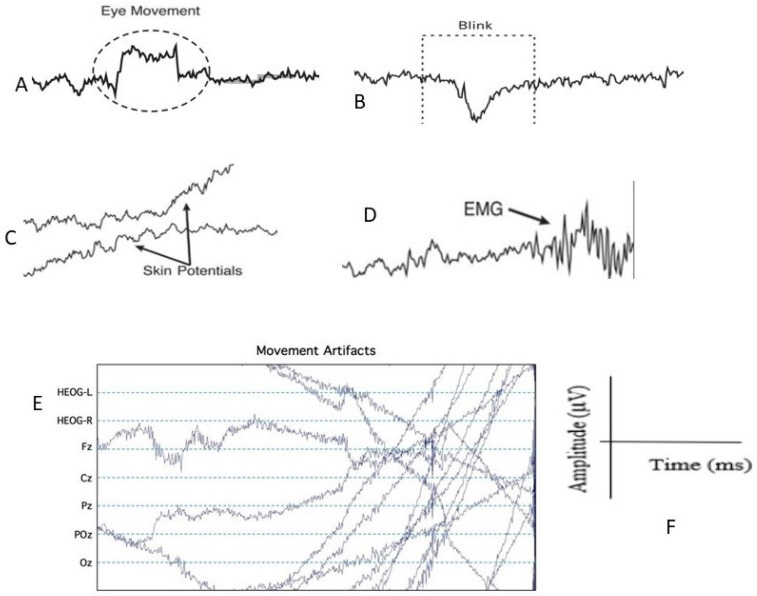
Example of common EEG artifacts that are easily identifiable by human visual inspection (Figures (**A**–**E**)). Figure (**A**) shows the eye-movement, (**B**) shows eye-blink, (**C**) shows skin potential, (**D**) shows EMG and (**E**) shows movement artifacts. Figure (**F**) indicates that the amplitude in microvolts is in the y-axis, and the x-axis shows the time in milliseconds [40].

The common artifacts (as shown in Figure 5) in the recorded EEG data are easy to identify. These identifications are performed based on the characteristics of the extracted features from the EEG data.

These corrupted artifacts and/or unfocused EEG data are different from neural EEG, and a proficient examiner (human) who has received training to recognize these abnormal characteristics in EEG data or ERP epochs can easily identify them by visual inspection. The following are examples [40,41]:

- Eye movements occur due to the prominent dipole within the eye. When the eyes remain still, this dipole generates a consistent voltage gradient across the scalp. However, when the eyes shift, the voltage increases in positivity on the side of the head toward which the eyes are directed (Figure 5A).
- When the eyes blink, the eyelid moves across the eyes, which leads to a large voltage of 200–400 ms and creates eye blink artifacts (Figure 5B).
- Skin potentials arise when sweat begins; it changes the impedance of the skin and it changes the standing electrical potential of the skin over a period of many seconds (as shown in Figure 5C).
- Muscle electrical potential created during the contraction of a muscle is called the electromyogram, or EMG. The EMG typically consists of rapid voltage fluctuations as shown in Figure 5D.
- Another very common artifact is due to body or head movement. In that case, the EEG follows no pattern. Sometimes the EEG might be in a positive or negative sloped pattern, sometimes it may zig-zag, etc. Figure 5E is one of the examples of movement artifacts.

For identifying unfocused EEG/ERP, the most effective approach is visual examination by humans. But this is not an efficient way as it is not only time-consuming but also boring. Thus, the replacement of this manual examination procedure is required, and it would be advantageous if the analysis could be performed automatically with good accuracy.

#### Analysis in the Lab by HVI

**Figure 6:**
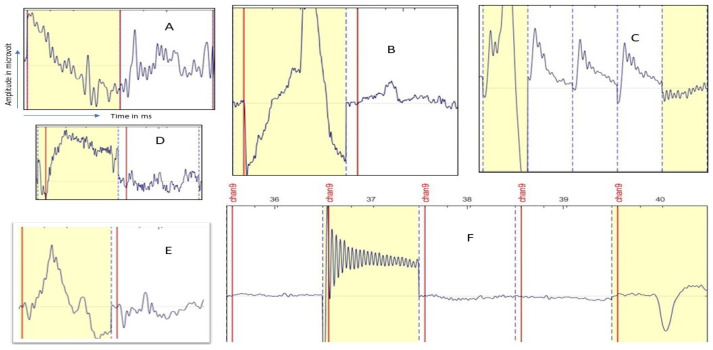
The figure shows an example of common EEG artifacts that are easily identifiable by human visual inspection. (**A**) shows the movement, (**B**,**C**) (first epoch) and (**F**) (last epoch) show eye-blink, (**C**) (last epoch) and (**F**) (second epoch) show EMG artifacts corrupted epochs. In every figure, the yellow-marked epoch is identified as the unusual or artifact corrupted epoch. The y-axis indicates the voltage amplitude (in microvolts) from the human scalp and the x-axis shows the time in milliseconds. These epoch figures are collected from Test Subjects 1 and 6.

Based on the identifying method as described in Section 6.1, in the normal laboratory conditions, we identified the following ERP epochs as unfocused or/and artifact-corrupted (marked by yellow color) as shown in Figure 6. For example, in Figure 6A, yellow-marked ERP epochs are considered unusual and/or movement artifacts. In Figure 6B,E, the yellow epochs are considered eye blink-corrupted epochs; in Figure 6C, the first yellow epoch also might be eye blink-corrupted; and the last one is considered either EMG or unusual compared to other ERP epochs. In Figure 6D, the yellow-marked epoch is considered as eye movement-corrupted and/or unusual ERP epochs. Finally, in Figure 6F, the first yellow-marked epochs are considered as muscle-corrupted artifacts (EMG), and the last one is also considered eye blink-corrupted.

### 6.2. Application of EEGLab and Analysis

**Figure 7:**
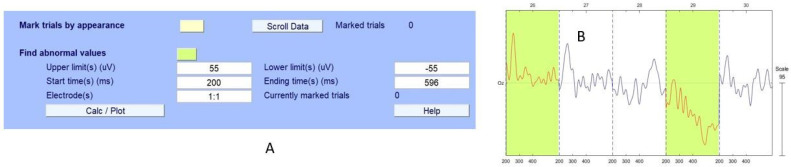
An example of common EEG artifacts that are easily identifiable by the EEGLab Toolkit. Figure (**A**) shows the EEGLab setup and Figure (**B**) shows the identified ERP epochs 26 and 29 (green marked) as artifact-corrupted and/or unusual.

We extracted the ERP epochs by EEGLab as was performed by HVI. EEGLab can effectively eliminate various non-brain artifacts, such as eye blinking and body/head movement. We established a threshold based on over-high amplitude levels of EEG epochs, setting it at 90% of the maximum peak amplitude within the period from −100 ms to 800 ms for detection. Figure 7A shows the EEGLab tool section for detecting abnormal amplitude levels. Also, Figure 7B shows how EEGLab identified corrupted epochs. Our analysis focused on the dataset pertaining to Test Subject 1, revealing that epochs 26 and 29 were marked by EEGLab as artifacts that are corrupted or unusual.

### 6.3. Application of Machine Learning Algorithms

In unsupervised learning, algorithms are responsible for identifying data patterns and associates inside the data deprived of prior guidance or any known knowledge [42,43]. They evaluate the data solely based on its inherent properties, grouping data points that are similar, by uncovering concealed decorations and associations. Additionally, machine learning algorithms utilize distances or densities as common techniques of estimation to distinguish between normal data and outliers [44,45].

For the identification of unusual ERP epochs, we applied Isolation Forest [46], Local Outlier Factor [47], DBScan [48], and k-means MLA techniques [49]. For the analysis by machine learning algorithms (MLAs), we used all default hyperparameters except the following:

- Contamination: The proportion of outliers in the dataset (default: 0.1). For our study, we set contamination = 0.2 for both Isolation Forest and the LOF.
- n_clusters: The number of clusters to form. The optimal value can be found using the elbow method, Silhouette score, or Gap statistic. We used the elbow method. For the k-means, we set n_clusters = 4, and all the other parameters were set to their default values.
- eps: The maximum distance between two samples for one to be considered in the neighborhood of the other (default: 0.5). The optimal value can be found using a k-distance graph. For DBScan, we set eps = 0.2.
- min_samples: The number of samples in a neighborhood for a point to be considered as a core point (default: 5). For DBScan, we set min_samples = 4.

The MATLAB tool was used for extracting the features from the ERP individual epochs. We extracted the following three features for the analysis by MLAs. They are as follows:

i. The peak amplitude of the EEG epoch (microvolt);
ii. The peak latency of the EEG epoch (millisecond);
iii. The mean value of the EEG epoch (microvolt).

**Figure 8:**
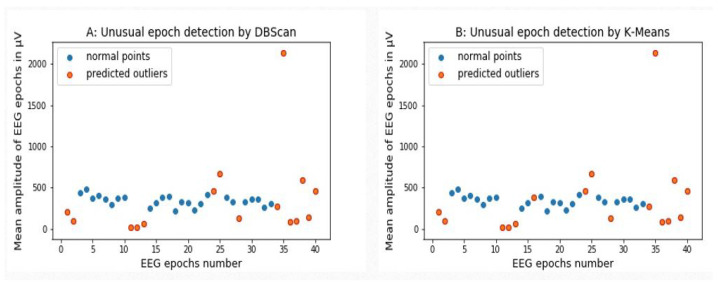
An example of common EEG artifacts detected by the DBScan (**A**) and k-means (**B**) MLA methods. In both figures, the unusual points are shown as orange circles, and the normal behavior data points are shown by blue circles.

Figure 8A illustrates a scatter plot depicting the application of the DBScan method in identifying abnormal EEG data within consistent EEG epochs. In this depiction, circles in the blue color represent consistent EEG epochs, while circles in the orange color denote abnormal epochs. Specifically, on the x-axis representing the epoch numbers, the unusual data points identified by DBScan are epochs 1, 2, 11, 12, 13, 24, 25, and 28 and epochs 34 to 40. Similarly, Figure 8B presents a scatter plot showcasing the k-mean method’s identification of abnormal ERP epochs (as shown in orange) contrasted with regular ERP epochs (blue) by their respective numbers.

## 7. Results and Analysis

**Figure 9:**
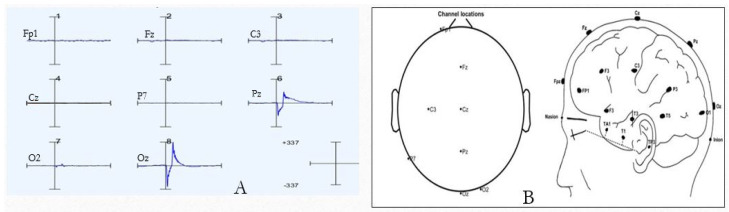
Figure (**A**) shows that the ERP epochs are larger and clearer in the position of the parietal (Pz) and occipital area (Oz). Figure (**B**) shows the electrode positions on the human scalp. We collected this dataset from Test Subject 1.

In this research, the EEG data were recorded from the eighth electrode positions of the human scalp from the frontal, central, parietal, and occipital areas (as shown in Figure 9). It was found that for almost all the participants, the ERP amplitude was greater for the parietal and occipital areas. P300 is the peak point of the ERP around 300 ms. P300 is often larger at parietal electrode sites because the parietal lobe processes attention, perception, and memory. Here, our subject needs to count uncommon stimuli with attention. On the other hand, the occipital lobe is primarily responsible for processing visual information.

For instance, if a task requires participants to discriminate between different visual stimuli or to pay attention to specific features of visual stimuli, then the occipital cortex may become more engaged. This increased involvement of the occipital cortex in processing visual information could lead to a larger P300 at occipital electrode sites, reflecting the enhanced neural activity associated with visual attention and discrimination tasks [50]. For the analysis of this study, the EEG data from the Oz position (as shown in Figure 9) were utilized.

### 7.1. Analyzing the ERP Epochs for the “Ideal” Experimental Condition

Table 4 shows the detected ERP epoch number as unfocused or/and artifact-corrupted by all the methods in which the datasets were collected from the “ideal” condition. From the table, it is shown that the ERP epoch numbers are not the same for every analyzing method. Several potential factors can contribute to the variation in the number of EEG epochs detected by different methods, such as human visual inspection, EEGLab, DBScan, k-means, Local Outlier Factor (LOF), and Isolation Forest, as observed in Table 4. For example, human inspectors may have different criteria for what constitutes an epoch, leading to variability. The level of expertise and experience of the inspector can affect detection accuracy. EEGLab offers various settings and preprocessing steps (e.g., filtering) that can influence the detection of epochs. Variations in the quality of the EEG signal (e.g., noise levels) can affect the performance of EEGLab in identifying distractions. On the other hand, every MLA has various parameters for which detection may vary. The parameters are discussed in Art 6.3.

From Table 4, it shows that HVI detected 4 epochs out of 50 epochs. Interestingly, the MLAs could sometimes detect more or less. For example, the Isolation Forest method detected six epochs, which is the maximum detection (nos. 1, 3, 9, 31, 32, and 35). On the other hand, EEGLab detected the minimum number of epochs (only nos. 32 and 35). The last column shows the unusual epoch detection accuracy in percentage (%) of all the analyzing methods compared to either HVI or epochs detected by the minimum of three MLAs among all four MLAs. The last row of the table shows the unusual epoch number, which was detected by a minimum of three MLAs among all four MLAs. From the accuracy results, it can be said that in the ideal condition, almost all the methods’ detection accuracy is very efficient with a range from 95% to 100%. DBScan shows perfect accuracy for the detection. The dataset for Test Subject 1 is used for all the analyses. For the ideal condition, HVI is considered the “gold standard” for unusual epoch detection.

**Figure 10:**
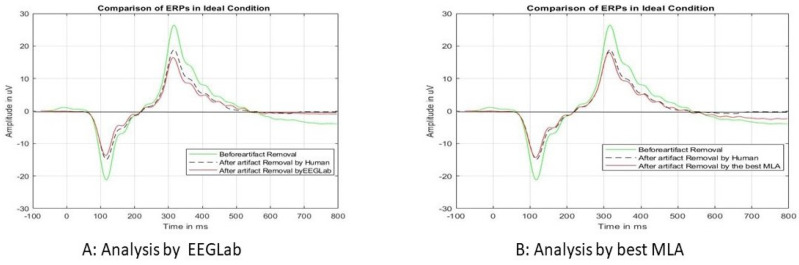
An analysis of the ERP epochs for the “ideal” condition. Figure (**A**) shows the comparison with HVI and Figure (**B**) shows the comparison with the best MLA (data for Test Subject 1).

Figure 10 shows the ERP epoch comparison before and after the unusual epochs are removed. In Figure 10A, the green-colored ERP represents the ERP plotted in the ideal condition. Then, the epochs (as in Table 4) were removed by HVI and plotted as black-dotted ERP and removed by EEGLab-marked epochs and plotted red ERP. In both cases, the ERP amplitude levels are reduced (17.28 microvolts by HVI vs. 19 microvolts by EEGLab), and there is no change in peak latency (324 ms for both).

In Figure 10B, the red ERP is the ERP where unusual epochs are removed by the best MLA (DBScan). The ERP amplitude is almost the same for both the HVI and MLA methods (17.09 microvolts by MLA and 17.28 microvolts by HVI) with the same peak latency of 324 ms. The reduction in ERP amplitude after removing unusual epochs has implications for signal quality, sensitivity, interpretability, consistency across studies, methodological considerations, and cautions in interpretation. By carefully addressing distractions and artifacts in EEG data preprocessing, researchers can obtain more reliable and valid insights into brain function and cognition, such as the following:

- The reduction in ERP amplitude after removing unusual epochs suggests that the unusual epochs contained noise or artifacts that interfered with the underlying neural signals. By removing these epochs, the signal-to-noise ratio of the EEG data is improved, resulting in cleaner and more reliable ERP waveforms.
- Removing unusual epochs may increase the sensitivity of the EEG analysis to detect subtle effects or differences in neural activity. By reducing the influence of noise and artifacts, the remaining epochs may better reflect the true underlying brain responses to the experimental manipulations or stimuli.
- Cleaner ERP waveforms can lead to more accurate and interpretable results in terms of the cognitive processes or neural mechanisms under investigation. Researchers can have greater confidence in the ERP effects observed in the data and their interpretation, knowing that they are less likely to be influenced by confounding factors, such as distraction-related artifacts.

To calculate the accuracy, a confusion matrix was used. The factors of the confusion matrix (Table 5) were defined as follows:

- True Positives (TPs): The number of epochs that are correctly predicted as unusual by either HVI or by at least three MLAs among all four MLAs.
- False Positives (FPs): The number of epochs that are incorrectly predicted as unusual by either HVI or by at least three MLAs among all four MLAs where it is not actually detected by either HVI or by at least three MLAs among all four MLAs.
- True Negatives (TNs): The number of epochs that are correctly predicted as not unusual by either HVI or by at least three MLAs among all four MLAs.
- False Negatives (FNs): The number of epochs that are incorrectly predicted as not unusual by either HVI or by at least three MLAs among all four MLAs where it is actually detected as unusual by either HVI or by at least three MLAs among all four MLAs.

### 7.2. Analyzing the ERP Epochs for the Visual Condition

Table 6 shows the detected ERP epoch number as unfocused or/and artifact-corrupted by all the methods for the “visual” experimental condition. Also, it is shown that HVI detected 10 ERP epochs out of 50. The Isolation Forest method detected 15 epochs with 95% accuracy, DBScan detected 13 epochs with perfect accuracy, k-means detected 10 epochs with 93% accuracy, and the LOF detected 13 epochs with 98% accuracy. On the other hand, EEGLab detected 10 epochs via HVI but with only 83% accuracy. The accuracy was measured as it was performed for the ideal condition. One point to be noted here is that, in ideal conditions, the HVI accuracy was 100%, but with visual distraction, the HVI accuracy was 88%.

If Table 5 and Table 6 are compared, then it shows that the accuracy of epoch detection in EEG research is significantly changed when transitioning from ideal (Table 5) experimental conditions to visual conditions. The reason might be that in ideal conditions, the environment is controlled to minimize artifacts and external distractions, making it easier to accurately identify unusual epochs. On the other hand, visual distractions might introduce more noise into the EEG recordings. This noise could come from both external sources and internal responses to distractions. Participants might likely have fluctuating attention levels, leading to more variability in the EEG signals. Visual distractions could cause indirect variations in the EEG data, such as changes in brain activity unrelated to the primary task but due to the distraction itself. Also, increased noise levels can lead to confusion, making it difficult to distinguish between actual epochs and unwanted noise.

HVI could overlook subtle variations in the typical characteristics of ERP, where these conventional MLAs proficiently identified additional epochs showing unusual behavior compared to standard epochs. Moreover, HVI is not considered the “gold standard” for detection in this context. MLAs can additionally address the focus level of test subjects by identifying abnormal epochs.

**Figure 11:**
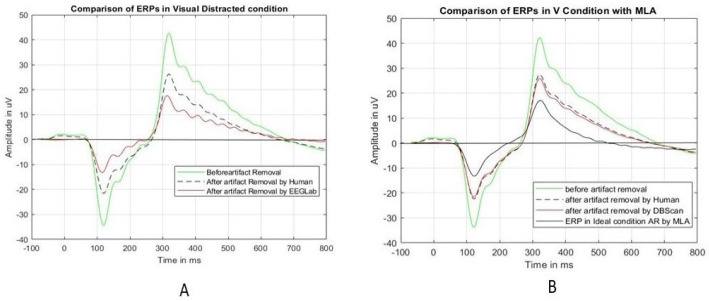
A comparison of the ERPs for the “visual” experimental condition for Test Subject 1. Figure (**A**) shows the comparison of HVI with EEGLab and Figure (**B**) shows the comparison of HVI with the best MLA.

Figure 11 shows the comparison before and after the unusual epochs are removed by HVI and EEGLab. The ERP before removing the unusual epochs is shown in the green color with a 43-microvolt peak amplitude and 326 ms peak latency. Then, the unusual epochs (as marked in Table 6) were removed by HVI, EEGLab, and the best MLA. Figure 11A shows the ERP after eliminating the unusual epochs by HVI (black dashed) and by EEGLab (red). Both the ERP peak amplitude and latency are reduced but with huge differences. The peak became 27.44 microvolts by HVI elimination and 18 microvolts by EEGLab elimination. Also, the peak latency became 324 ms and 311 ms, respectively. Figure 11B shows the ERP (red) after eliminating the unusual epochs by DBScan with a peak of 26 microvolts at 324 ms. Interestingly, this is almost the same as that eliminated by HVI. The black solid line ERP shows the ideal condition.

### 7.3. Analyzing the ERP Epochs for the “Audio–Visual” Experimental Condition

Table 7 shows the detected ERP epoch number as unfocused or/and artifact-corrupted for the “audio–visual” condition for the same dataset. It shows that HVI detected 11 ERP epochs out of 50 epochs as unusual, with 85% accuracy.

The Isolation Forest method detected 15 epochs with 98% accuracy, DBScan detected 15 epochs with perfect accuracy, k-means detected 16 epochs with 98% accuracy, and the LOF detected 15 epochs with 98% accuracy. On the other hand, EEGLab detected the minimum number of epochs (only eight) as epochs with only 78% detection accuracy. Like the “visual distraction” condition, the accuracy for HVI (85%) is not as perfect as it was in the ideal condition, but the ML methods are all efficient with an accuracy between 95% and 100%.

**Figure 12:**
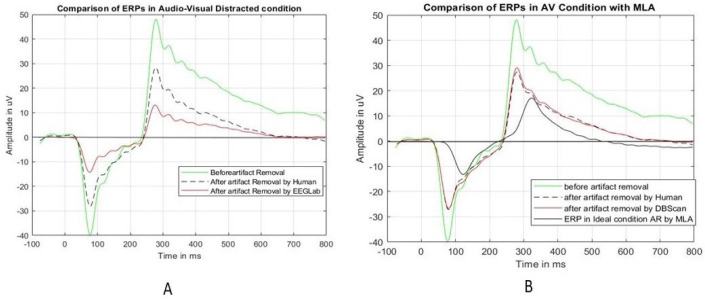
A comparison of the ERPs for the “audio-visual” condition for Test Subject 1. Figure (**A**) shows the comparison of HVI with EEGLab and Figure (**B**) shows the comparison with the best MLA.

Figure 12 shows the comparison of the ERP epochs before and after the unusual epochs are removed by HVI and EEGLab in the AV condition. The ERP before removing the unusual epochs is shown in the green color with a 48-microvolt peak amplitude and 286 ms peak latency. Then, the unusual epochs (as marked in Table 7) were removed by HVI, EEGLab, and the best MLA. Figure 12A shows the ERP after eliminating the unusual epochs by HVI (black dashed) and EEGLab (red). Both the ERP peak amplitude and latency are reduced but with huge differences. The peak became 27 microvolts by HVI elimination and 14 microvolts by EEGLab elimination. Also, the peak latency became 286 ms and 311 ms, respectively. On the other hand, Figure 12B shows the ERP (red) after eliminating the unusual epochs by DBScan with a peak of 27.44 microvolts at 296 ms. Interestingly, this is almost the same as that eliminated by HVI.

If we compare Figure 11 (ERP comparison with visual distraction) with Figure 12, it shows that there are differences in the ERP peaks. The difference in the ERP peak might arise due to the additional audio distraction. For example, audio distractions may elicit varying neural responses, the timing and duration of audio distractions can affect ERP measurements differently, audio distractions may engage attentional resources to varying degrees, etc.

### 7.4. Analyzing the ERP Epochs for the “Mind-Wandering” Experimental Condition

Table 8 shows the detected ERP epoch number as unfocused or/and artifact-corrupted by all the methods in which datasets were collected from the “mind-wandering (MW)” experimental condition. As described in previous tables, the last column of Table 8 shows the unusual epoch detection accuracy in percentage (%) of all the analyzing methods compared to either HVI or epochs detected by the minimum of three MLAs among all four MLAs. The last row of the table shows the unusual epoch number, which was detected by a minimum of three MLAs among all four MLAs. Table 8 shows that HVI detected 13 ERP epochs out of 50 epochs as unusual with 98% accuracy. Remarkably, the MLAs could detect more for all four MLAs. For example, Isolation Forest, DBSCan, and k-means were all the methods that detected 15 epochs (not same) with 98% accuracy. On the other hand, the LOF detected 14 epochs with perfect accuracy. EEGLab detected 14 epochs as unusual epochs with only 78% detection accuracy. Like other conditions, HVI is not the ideal method for unusual epoch detection, and MLAs are better at comparing two, such as HVI and EEGLab. MLAs can additionally address the focus level of test subjects by identifying abnormal epochs.

**Figure 13:**
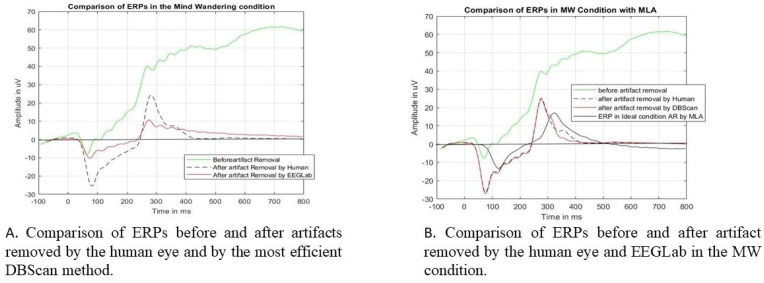
An analysis of the ERP epochs for the “mind-wandering” condition. Figure (**A**) shows the comparison with HVI and Figure (**B**) shows DBScan. The black solid-line ERP is the ERP in the ideal condition after the artifact was corrupted or the epochs were removed by HVI.

Figure 13 shows the ERP epoch comparison before and after the unusual epochs are removed from the (mind-wandering) dataset by HVI and EEGLab. The ERP before removing the unusual epochs is shown in the green color. It shows a very abnormal ERP pattern. Then, the unusual epochs (as marked in Table 8) were removed by HVI (black dashed), EEGLab, and the best MLA. From Figure 13A, it is shown that after eliminating the unusual epochs by HVI (black dashed) and EEGLab (red), both the ERP peak amplitude and latency are reduced but with huge differences. The peak became 24 microvolts by HVI elimination and 10 microvolts by EEGLab elimination.

Also, the peak latency became 286 ms and 275 ms, respectively. On the other hand, Figure 13B interestingly shows that after eliminating the unusual epochs by DBScan, the ERP (red) peak becomes 23.8 microvolts at 286 ms, which is almost the same as that eliminated by HVI. The black solid-line ERP shows the ideal condition.

## 8. Discussion

This dissertation explores the utilization of unsupervised machine learning processes for the screening of ERP data epochs. Specifically, it investigates their capability to recognize and filter out data epochs exhibiting unfamiliar characteristics compared to consistently natured epochs. These ERP epochs may be affected by signal degradation, artifacts (such as eye blinks, body movements, EMG, etc.), or distractions or a lack of focus from the test subjects. Unsupervised MLAs are used in the research to analyze EEG epochs in a P300 experiment of the oddball paradigm. In a typical electronic systems investigation, four experimental setups (ideal, distraction by sound, distraction by both sound and visual, and mind wandering) were used to collect data. Our goal was to look at EEG epochs during times of distraction and see how they affected things. In the last phase, which was deemed to be the optimal setting, the participants were told to move as little as possible to gather EEG data as intently as possible.

The predictable method for analyzing ERP data includes recognizing and removing epochs corrupted by regular EEG artifacts, like eye blinks, eye movements, and body or muscle movements. In this work, unsupervised MLAs, EEGLab, and human visual inspection (HVI) were used to evaluate the ERP epochs. Although it is generally considered that HVI is the gold standard for classification, our findings indicate that it may be able to miss small variations in common ERP patterns. When distraction or mind-wandering behaviors were present in the experimental circumstances, we saw a greater frequency of epochs with minor variations. Furthermore, several widely used unsupervised MLAs were able to identify anomalous epochs in contrast to regular ones. We defined a threshold where atypical or artifact-corrupted epochs are recognized by HVI or by at least three of the four MLAs evaluated in this research in order to simplify a comparison between HVI and unsupervised MLAs.

As per the established norm, DBScan demonstrated the maximum accuracy, with results ranging from 98% to 100% when it came to recognizing uncommon and artifact-corrupted epochs. In contrast, under experimental conditions involving mind wandering, visual distraction, or audio/visual distraction, human visual inspection demonstrated varied accuracy, ranging from 85% to 98%. With accuracy ranging from 78% to 83% under identical experimental settings, the EEGLab Toolkit had the lowest accuracy. Furthermore, the remaining data produced ERP waves that were quite similar to those recorded under the “ideal” data collecting setting after all the identified anomalous epochs were eliminated from the dataset.

While the unsupervised MLAs enable improvement in the ability to capture and analyze ERP data collected under non-ideal conditions, the dataset was insufficient to allow for a detailed analysis of how distraction impacts ERP data. Determining what is happening in the brain when distraction is present in the ERP experiment would require more rigorous experiments that more carefully control for distraction and assess the actual level of distraction that a test subject experiences. While conducting this research, this work had some limitations. For example, this study was unable to measure the test subjects’ level of distraction independently. Moreover, this research was unable to regulate how the test subjects’ distraction was changed. For obtaining more accuracy, the effect of distraction should be assessed more precisely. Also, changing the experimental setting, increasing the number of test subjects, performing some psychological tests that can measure the degree of distraction, etc., can be added to this study for further research.

## Figures and Tables

**Table 1:** Source and types of EEG artifacts [24].

Artifacts	Types	Source
Eye Blinks, Eye Movements	Ocular	Biological
Chewing, Swallowing, etc.	Muscular	Biological
Electrocardiogram	Cardiac	Physiological
Skin Response, Sweltering	Skin	Biological
Power Line	Electrical/electronics	Physiological
Head and Body Movement	Movement of the body	Biological

**Table 2:** The 4 experimental setups for this study.

Experimental Sequence	Setup Sequence	Duration
Step-0	System setup and test subjects were introduced to the common and odd tones	5–10 min
Step-1: Mind Wandering	Subject heard the tones without any instruction	3–4 min
	Rest	4–5 min
Step-2: Visual Distraction	Subject heard the tones and counted the odd stimuli while watching a movie without sound	3–4 min
	Rest	4–5 min
Step-3: Audio–Visual Distraction	Subject heard the tones and counted the odd stimuli while watching a movie with sound	3–4 min
	Rest	4–5 min
Step-4: Ideal Condition (Final Step)	Subject heard the tones and counted the odd stimuli giving maximum focus, making less or no movement	3–4 min

**Table 3:** Three methods of analysis for the identification of unfocused epochs.

Steps for Data Analysis	Ideal Method Dataset G	Test Method 1 Dataset Tx	Test Method 2 Dataset Tx	Test Method 3 Dataset Tx
Raw EEG Data Collection	Data Collection by Step 4	Data Collection by Steps 1, 2, and 3	Data Collection by Steps 1, 2, and 3	Data Collection by Steps 1, 2, and 3
Dividing Epochs	EEG Lab	EEG Lab	EEG Lab	EEG Lab
Artifact Detection	HVI	HVI	EEG Lab	MLA
Averaging of EEG Epochs	EEG Lab	EEG Lab	EEG Lab	EEG Lab
Plotting ERP and Analysis	EEG Lab	EEG Lab	EEG Lab	EEG Lab

x = 1, 2, 3.

**Table 4:** The evaluation of the accuracy in identifying unusual and/or artifact-corrupted ERP epochs between EEGLab and MLAs via human visual inspection (HVI) for the “ideal” setup condition.

Analyzing Method	Unusual ERP Epoch Number						Comparing Accuracy with Human or Minimum 3 of the 4 MLAs
HVI	1	–	–	31	32	35	100%
EEGLab	–	–	–	–	32	35	95%
Isolation Forest	1	3	9	31	32	35	95%
DBScan	1	–	–	31	32	35	100%
K-Means	1	–	–	31	32	–	98%
LOF	1	3	–	31	32	35	98%
Epochs detected by 3/4 MLAs	1	–	–	31	32	35	

**Table 5:** Confusion matrix.

	Actually Positive	Actually Negative
Predicted Positive	TP	FP
Predicted Negative	FN	TN

**Table 6:** The evaluation of the accuracy in identifying unusual and/or artifact-corrupted ERP epochs using both EEGLab and MLAs compared to human visual inspection (HVI) for the “visual” condition.

Analyzing Method	Unusual ERP Epoch Number																Comparing Accuracy with Human or Minimum 3 of the 4 MLAs
HVI	1	2	3	–	–	–	–	16	23	25	26	32	–	–	39	40	88%
EEGLab	1	–	3	4	5	6	7	16	–	25	–	–	–	–	39	40	83%
Iso.Forest	1	2	3	4	5	6	7	16	–	25	26	32	37	38	39	40	95%
DBScan	1	2	3	4	5	6	7	16	–	25	26	32	–	–	39	40	100%
K-Means	1	2	3	4	5	–	–	–	–	25	26	32	–	–	39	40	93%
LOF	1	2	3	4	5	6	7	16	–	25	26	32	–	–	39	40	98%
Epo-det by 3/4 MLAs	1	2	3	4	5	6	7	16	–	25	26	32	–	–	39	40	

Epo-det: epochs detected.

**Table 7:** The evaluation of the accuracy in identifying unusual and/or artifact-corrupted ERP epochs between EEGLab and MLAs with human visual inspection (HVI) for the “audio–visual ” condition.

Analyzing Method	Unusual ERP Epoch Number																	Comparing Accuracy with Human or Minimum 3 of the 4 MLAs
HVI	-	2	-	11	12	13	16	-	-	-	-	35	36	37	38	39	40	85%
EEGLab	-	2	3	-	-	-	-	-	25	-	34	35			38	39	40	78%
Iso.For	1	2	-	11	12	13	-	24	25	28	34	35	36	37	38	39	40	98%
DBS	1	2	-	11	12	13	16	24	25	28	34	35	36	37	38	39	40	100%
K-Means	1	2	-	11	12	13	-	24	25	28	-	35	36	37	38	39	40	95%
LOF	1	2	-	11	12	13	-	24	25	28	34	35	36	37	38	39	40	98%
Epo-det by 3/4 MLAs	1	2	-	11	12	13	-	24	25	28	34	35	36	37	38	39	40	

Last column: Comp.Accu with Hum. means “comparing accuracy with human”. Epo-det: epochs detected.

**Table 8:** The evaluation of the accuracy in identifying unusual and/or artifact-corrupted ERP epochs between EEGLab and MLAs with human visual inspection (HVI) for the “mind-wandering” condition.

Analy. Method	Unusual ERP Epoch Number																			Acc. Comp.
HVI	1	2	3	4	5	6	7	8	13	-	-	-	-	-	32	-	38	39	40	98%
EEGLab	-	-	-	-	5	6	7	8	13	23	24	25	26	-	32	33	38	39	40	78%
IF	1	2	3	4	5	6	7	8	13	-	-	-	-	31	32	33	38	39	40	98%
DB	1	2	3	4	5	6	7	8	13	23	-	-	-	31	32	-	38	39	40	98%
KM	1	2	3	4	5	6	7	8	13	23	-	-	-	31	32	-	38	39	40	98%
LOF	1	2	3	4	5	6	7	8	13	-	-	-	-	31	32	-	38	39	40	100%
Epo-det by 3/4 MLAs	1	2	3	4	5	6	7	8	13	-	-	-	-	31	32	-	38	39	40	

IF: Isolation Forest; DB: DBScan; KM: k-means; and Epo-det: epochs detected. Last Column: Acc. Comp. means “comparing accuracy with human or minimum 3 of the 4 MLAs”.

## Data Availability

The data was recorded in the Lab of UGA and it is not publicly available.
